# Retraction Note to: FOXM1 modulates 5-fluorouracil sensitivity in cholangiocarcinoma through thymidylate synthase (TYMS): implications of FOXM1–TYMS axis uncoupling in 5-FU resistance

**DOI:** 10.1038/s41419-021-03807-4

**Published:** 2021-05-25

**Authors:** Kitti Intuyod, Paula Saavedra-García, Stefania Zona, Chun-Fui Lai, Yannasittha Jiramongkol, Kulthida Vaeteewoottacharn, Chawalit Pairojkul, Shang Yao, Jay-Sze Yong, Sasanan Trakansuebkul, Sakda Waraasawapati, Vor Luvira, Sopit Wongkham, Somchai Pinlaor, Eric W.-F. Lam

**Affiliations:** 1 Department of Surgery and Cancer, Imperial College London, Imperial College London, Hammersmith Hospital Campus, London, W12 0NN UK; 2 Biomedical Science Program, Graduate School, Khon Kaen University, Khon Kaen, 40002 Thailand; 3 Cholangiocarcinoma Research Institute, Khon Kaen University, Khon Kaen, 40002 Thailand; 4 Department of Biochemistry, Faculty of Medicine, Khon Kaen University, Khon Kaen, 40002 Thailand; 5 Department of Pathology, Faculty of Medicine, Khon Kaen University, Khon Kaen, 40002 Thailand; 6 Department of Surgery, Faculty of Medicine, Khon Kaen University, Khon Kaen, Thailand; 7 Department of Parasitology, Faculty of Medicine, Khon Kaen University, Khon Kaen, Thailand

**Keywords:** Cancer therapy, Transcription

Retraction Note to: *Cell Death & Disease*

10.1038/s41419-018-1235-0 published online 11 December 2018

The Editors-in-Chief have retracted this article at the request of Imperial College London. An investigation concluded that there are a number of concerns with this article, specifically:

- Figure 2B represented technical repeats, and not biological repeats as is implied by the legend.
- Figure 4A: the second and third panel representing results of the KKU-D131 cells have been altered to bring them more in line with other test results, though this was not declared in the article. Additionally, similar concerns were also raised regarding the 5th and 6th bars in the E2F1 mRNA and TYMS mRNA panels.
- Figure 7B: the underlying data for this figure has been manipulated.
- The article suggests that the Western blots and RT-qPRC results in Figures 4 and 7 came from contemporaneous experiments. However, the investigation found that the western blotting and the mRNA determinations had been performed by different researchers more than a year apart.

Paula Saaveedra Garcia and Chun-Fui Lai agree with this retraction. Eric Lam, Stafania Zona, Kulthida Vaeteewoottacharn, Chawalit Pairojkul, Sakda Waraasawapati, Vor Luvira, Sopit Wongkham, Somchai Pinlaor and Kitti Intuyod disagree with this retraction. Shang Yao, Jay-Sze Yong, Sasanan Trakansuebkul and Yannasittha Jiramongkol have not responded to correspondence regarding this retraction.

